# African KhoeSan ancestry linked to high-risk prostate cancer

**DOI:** 10.1186/s12920-019-0537-0

**Published:** 2019-06-04

**Authors:** Desiree C. Petersen, Weerachai Jaratlerdsiri, Abraham van Wyk, Eva K. F. Chan, Pedro Fernandez, Ruth J. Lyons, Shingai B. A. Mutambirw, Andre van der Merwe, Philip A. Venter, William Bates, M. S. Riana Bornman, Vanessa M. Hayes

**Affiliations:** 1 Laboratory for Human Comparative and Prostate Cancer Genomics, Genomics and Epigenetics Division, Garvan Institute of Medical Research, The Kinghorn Cancer Centre, 370 Victoria Street, Darlinghurst, NSW 2010 Australia; 2 Faculty of Medicine, University of New South Wales, Randwick, NSW Australia; 3 Division of Anatomical Pathology, NHLS Tygerberg and Stellenbosch University, Tygerberg, South Africa; 4 Division of Urology, Department of Surgical Sciences, Faculty of Medicine and Health Sciences, Stellenbosch University, Tygerberg, South Africa; 5 Department of Urology, Sefako Makgatho Health Science University, Dr George Mukhari Academic Hospital, Medunsa, South Africa; 6 Faculty of Health Sciences, University of Limpopo, Mankweng, South Africa; 7 School of Health Systems and Public Health, University of Pretoria, Pretoria, South Africa; 8 Central Clinical School, University of Sydney, Camperdown, NSW Australia; 9 Centre for Proteomic and Genomic Research (CPGR), 1st Floor, St. Peters Mall, Cnr. Anzio and Main Road, Observatory, Cape Town, 7925 South Africa

**Keywords:** African ancestry, Prostate cancer, KhoeSan, High-risk disease, Ancestral fractions, Ancestry informative markers

## Abstract

**Backgrounds:**

Genetic diversity is greatest within Africa, in particular the KhoeSan click-speaking peoples of southern Africa. South African populations represent admixture fractions including differing degrees of African, African-KhoeSan and non-African genetic ancestries. Within the United States, African ancestry has been linked to prostate cancer presentation and mortality. Together with environmental contributions, genetics is a significant risk factor for high-risk prostate cancer, defined by a pathological Gleason score ≥ 8.

**Methods:**

Using genotype array data merged with ancestry informative reference data, we investigate the contribution of African ancestral fractions to high-risk prostate cancer. Our study includes 152 South African men of African (Black) or African-admixed (Coloured) ancestries, in which 40% showed high-risk prostate cancer.

**Results:**

Genetic fractions were determined for averaging an equal African to non-African genetic ancestral contribution in the Coloured; we found African ancestry to be linked to high-risk prostate cancer (*P-value =* 0.0477). Adjusting for age, the associated African ancestral fraction was driven by a significant KhoeSan over Bantu contribution, defined by Gleason score ≥ 8 (*P-value =* 0.02329) or prostate specific antigen levels ≥20 ng/ml (*P-value =* 0.03713). Additionally, we observed the mean overall KhoeSan contribution to be increased in Black patients with high-risk (11.8%) over low-risk (10.9%) disease. Linking for the first time KhoeSan ancestry to a common modern disease, namely high-risk prostate cancer, we tested in this small study the validity of using KhoeSan ancestry as a surrogate for identifying potential high-risk prostate cancer risk loci. As such, we identified four loci within chromosomal regions 2p11.2, 3p14, 8q23 and 22q13.2 (*P-value =* all age-adjusted < 0.01), two of which have previously been associated with high-risk prostate cancer.

**Conclusions:**

Our study suggests that ancient KhoeSan ancestry may be linked to common modern diseases, specifically those of late onset and therefore unlikely to have undergone exclusive selective pressure. As such we show within a uniquely admixed South African population a link between KhoeSan ancestry and high-risk prostate cancer, which may explain the 2-fold increase in presentation in Black South Africans compared with African Americans.

**Electronic supplementary material:**

The online version of this article (10.1186/s12920-019-0537-0) contains supplementary material, which is available to authorized users.

## Background

High-risk prostate cancer (HRPCa) accounts for approximately 15% of diagnoses in Western countries, with significant potential for associated lethality [1]. Although a number of HRPCa classifications have been proposed, including variations in the requirement for clinical tumor staging and serum prostate specific antigen (PSA) levels, HRPCa is typically defined as pathological Gleason score (GS) ≥ 8 or PSA ≥ 20 ng/ml at diagnosis. In the United States, African American men are disproportionally affected by HRPCa. Overall, mortality rates are 2-fold higher in American men of African versus European ancestry, while reaching as much as a 4.2-fold increase among younger men [2]. Further support for a bias towards aggressive prostate cancer presentation within African American men includes: elevated PSA levels and younger age at diagnosis, a shorter PSA doubling time prior to surgery, higher tumor grade and volume at surgery, higher rates of biochemical relapse post-surgery and reduced rates of curative therapy [3].

HRPCa is also disproportionally observed in men from sub-Saharan Africa and Southern Africa [4, 5]. Compared with African Americans, the latter study has showed Black South African men are at a 2.1-fold and 4.9-fold greater risk for presenting at diagnosis with GS ≥ 8 and PSA ≥ 20 ng/ml, respectively. While socioeconomic and lifestyle factors, as well as late detection contribute to the disproportionate impact of HRPCa within African Americans, data within Africa is severely lacking. We have previously discussed social, cultural, educational and economic factors contributing to advanced disease presentation within southern Africa, while highlighting the unique opportunity the region holds for leveraging new knowledge with regards to prostate cancer risk and biology, including genetic contribution [6]. The significance of genetic contribution to HRPCa cannot be ignored [3, 7].

In addition to significant HRPCa presentation in Black South Africans [5], HRPCa is also elevated within the African-admixed population from South Africa, the South African Coloured [5, 8]. While Black South Africans represent a uniquely African ancestry, predominantly Bantu, with contributing KhoeSan heritage, the Coloured arose as a result of intermarriage between initial European colonists, Dutch East Indian slaves and indigenous Bantu and KhoeSan Southern Africans [9, 10]. Therefore, the genetic ancestral fractions of the South African Coloured uniquely represent the broad spectrum of prostate cancer racial disparity reported in the United States, specifically African-biased high-risk, European-biased intermediate-risk (GS = 7) and Asian-biased low-risk prostate cancer (LRPCa; GS = 6). In this study we determine if African ancestry, specifically Bantu or KhoeSan African ancestry, is preferentially linked to HRPCa presentation in the region.

## Methods

### Study participants

South African men self-identifying as Black (*n* = 68) or Coloured (*n* = 84) presented at the urology clinics at Polokwane (Limpopo Province), Steve Biko or Dr. George Mukhari (Gauteng Province) or Tygerberg (Western Cape Province) Academic Hospitals. The study was approved and participants consented as required by local ethics approvals, with participant recruitment within Limpopo and Gauteng as part of the previously described Southern African Prostate Cancer Study (SAPCS) [5, 11]. DNA was extracted from whole blood using standard methods (QIAGEN Inc., Germantown, Maryland) and deidentified samples shipped to Australia for further genomic analyses (refer to ethics approvals and permits).

### Clinical and pathological presentation

Presence or absence of prostate cancer was provided by clinicopathological diagnosis. All biopsy cores underwent independent rescoring for the 50 Black cases and 18 Black cancer-free patients as previously described [12] and the 84 Coloured cases (by AvW and WB, see Additional file 1). HRPCa defined as a GS ≥ 8, was confirmed for 33 Black (66%) and 27 Coloured (32%), or PSA ≥ 20 ng/ml (irrespective of pathological features), was observed for 36 Black (72%) and 39/81 Coloured (48%). LRPCa defined as a GS = 6, was observed for seven Black (14%) and 12 Coloured (14%), or PSA < 10 ng/ml for six Black (12%) and 23 Coloured (28%). The remaining patients were classified as presenting with intermediate risk disease.

### Genomic data generation

Illumina Infinium HumanCore Beadchip (> 250 K markers) genotype array data was either made available (68 Black) [12] or generated (84 Coloured). Data inclusion was dependant on a GenTrain score (a measure representing the reliability of the genotype calls) of at least 0.5 or more (Illumina GenomeStudio 1.9.4) with further selection of autosomal markers based on a linkage disequilibrium r^2^ value > 0.2 within a 50-variant sliding window, advanced by five variants at a time (SNP and Variation Suite 8.3.1, Golden Helix).

### Determining ancestral fractions

Genomic data from population representatives (in brackets) for different African ancestral identifiers were used and defined as: KhoeSan (Ju/‘hoansi) [9], West African (Mandinka), Proto-Bantu (Yoruba), West Bantu (Bamoun and Fang), and East Bantu (Luhya) [13], while non-African ancestral identifiers included: Asian (Han Chinese) and European (Utah Americans) (Illumina iControl data). African American data (*n* = 48) was sourced from the International Genome Sample Resource. Ancestral fractions were estimated using STRUCTURE 2.3.3 (5000/10000 burn-in iterations, 10,000/20000 replicates) assuming different ancestral contributions (≥ five replications) [14].

### Statistical analyses

Statistical analyses were performed in R (https://www.r-project.org) using linear regression (lm) of continuous or categorical data. One-way ANOVA was used for establishing significant disease predictors. Two tailed t-test was used to determine an association between African ancestry and risk extremes, namely HRPCa versus LRPCa. RFMix analysis for local ancestry inference was used to estimate admixture across 22 individual pairs of autosomes [15]. Genotyping data of 84 Coloured patients were removed if unmapped to GRCh37, and phased using SHAPEIT2 with the 1000 Genomes Phase 3 reference panel [16]. RFMix was run with two expectation maximization iterations and 0.2 centimorgan (cM) window size and results of each patient along with the population representatives described above were converted to genomic intervals with ancestral identifiers. The intervals where KhoeSan contributions between HRPCa and LRPCa (defined by either GS or PSA) differed greater than three times were compared using Fisher’s exact significance test and then Bonferroni correction (46 and 45 intervals compared based on GS and PSA values, respectively). Significant phased intervals greater than one megabase were chosen for single marker and haplotype block association tests using Haploview (https://www.broadinstitute.org/haploview/haploview). The RFMix results with posterior probability greater than 0.9 were modelled for migration timing and gene flow estimation using the ancestry tracts analysis (TRACTS) program [17]. The best-fit model assuming KhoeSan, Bantu and Eurasian contributions, was selected based on likelihood values.

## Results

### Population specific ancestral fractions

**Fig. 1:**
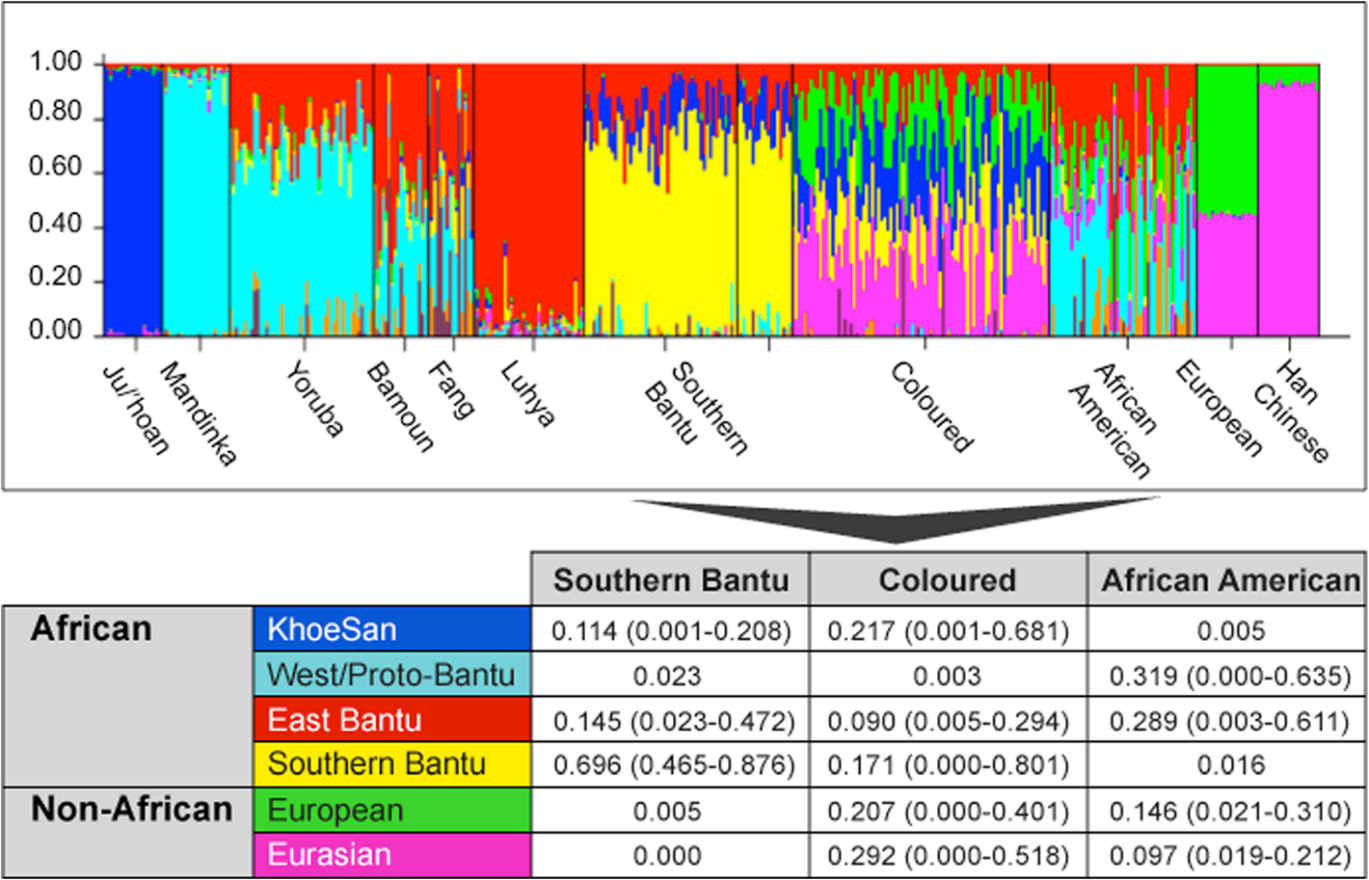
Population substructure of the study participants. (Top Panel) STRUCTURE analysis for 10,295 autosomal markers for eight ancestral populations and including the 68 Black (50 cases and 18 controls) and 84 Coloured South African (SA) study participants compared with African Americans and reference populations from Africa (Ju/‘hoansi, Mandinka, Yoruba, Bamoun, Fang and Luhya) and outside of Africa (European and Han Chinese) for a total of 397 subjects. (Middle Panel) Using STRUCTURE analysis we determined the African ancestral fractions, defined as KhoeSan, West/Proto-Bantu, East Bantu and Southern Bantu, as well as the non-African ancestral fractions, defined as European and Eurasian, within our study cohort with comparisons made with the African Americans

STRUCTURE analysis using 10,295 autosomal markers provided detailed population substructure (Fig. 1 based on eight reference populations). In contrast to African Americans, the African ancestral contributions to the study participants are almost exclusively Bantu and KhoeSan. While African Americans lack KhoeSan contributions, their African ancestral contribution is largely West African (non-Bantu with a lesser West/Proto-Bantu contribution) and East Bantu, with a significant European-biased non-African contribution. The Bantu contribution in our study participants can be defined as uniquely Southern Bantu, 69.6% in the Black and 17.1% in the Coloured, with a smaller East Bantu fraction, 14.5 and 9%, respectively. KhoeSan contributions range from minimal up to 20.8% in the Black and as much as 68.1% in the Coloured.

**Fig. 2:**
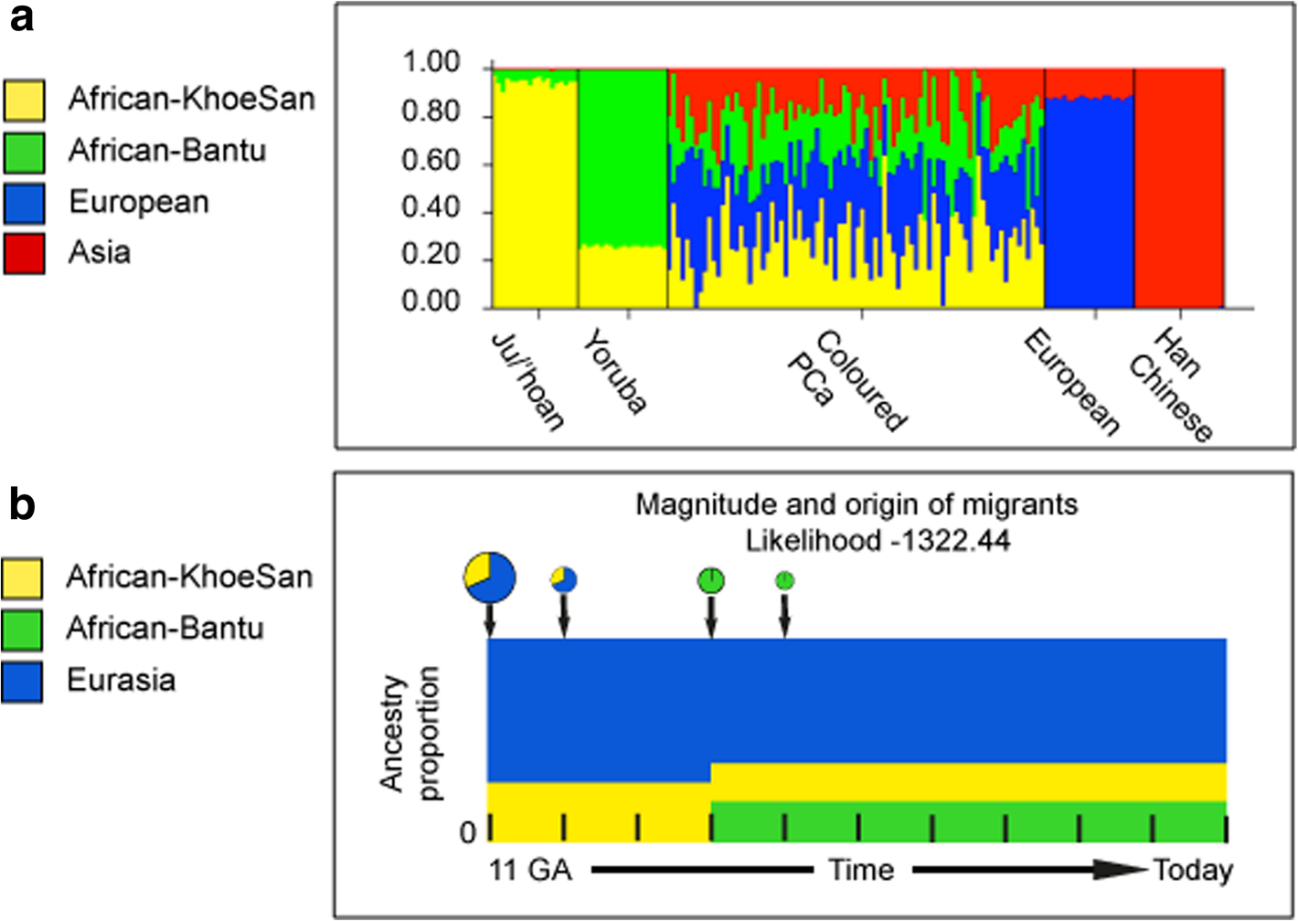
**a** Ancestral fractions determined using STRUCTURE analysis 84 South African Coloured men with PCa using 114,199 autosomal markers and K = 4 (5000 burn in and 10,000 reps) identifying ancestral contributions defined as African-KhoeSan, African-Bantu, European and Asian. **b** Magnitude and origin of migrants is shown with different colors in bar and pie charts representing three ancestral contributions. The size of pie charts is proportional to percentage of migrants, with the earliest generation equal to 100% and a decrement in the next generation

While the Black participants show exclusive African heritage, the Coloured present overall with an almost equal non-African to African fraction. A 9-fold increase in the number of ancestry informative markers through limiting founder population inclusion (91,263 markers) allowed for further separation of the non-African Coloured fractions into European (range 0 to 62.3%) and Asian (range 0. 3 to 42.2%) (Fig. 2a). To better understand the extent of African ancestral contributions in our Coloured participants (*n* = 84), we used TRACTS to model their migration history. Consequently, we defined the Coloured as migratory non-African, with significant KhoeSan contributions from 11 (31.5%) to 10 (7.1%) generations ago, followed by Bantu contributions appearing 8 (20.4%) and 7 (11.8%) generations ago (Fig. 2b). In contrast, the KhoeSan contribution to the Black population (*n* = 68) appeared as a single pulse migration event roughly 21 generations ago (11.1%; Optimal likelihoods value: − 255.7).

### African ancestral fractions linked to HRPCa

Presenting with an almost even distribution of African to non-African heritage, the Coloured provide an ideal genetic resource to further evaluate the African ancestral contribution to HRPCa. We observed a significant association between total African ancestry and prostate cancer pathology. Participants with HRPCa (GS ≥ 8) showed an average of 54.8% African ancestry compared to the 37.3% observed for patients with LRPCa (GS = 6) (t = 2.0974, *P*-value = 0.0477). Furthermore, we observed a significant KhoeSan over Bantu African contribution to HRPCa, specifically the average KhoeSan contributions to GS ≥ 8 versus 6 tumors was 31 and 20.1%, respectively (t = 2.4491, *P*-value = 0.0233) and for PSA ≥ 20 versus < 10 ng/ml tumors, 31 and 24.1%, respectively (t = 2.1455, *P*-value = 0.0371). Although the total KhoeSan contribution to the Black patients was less significant (range 0 to 21%), we did note a slight increase in total KhoeSan ancestral contribution within patients presenting with GS ≥ 8 versus 6 tumors (mean 11.8% vs 10.9%; t = 0.3249, *P*-value = 0.754).

### HRPCa loci enriched for KhoeSan ancestral contribution

**Fig. 3:**
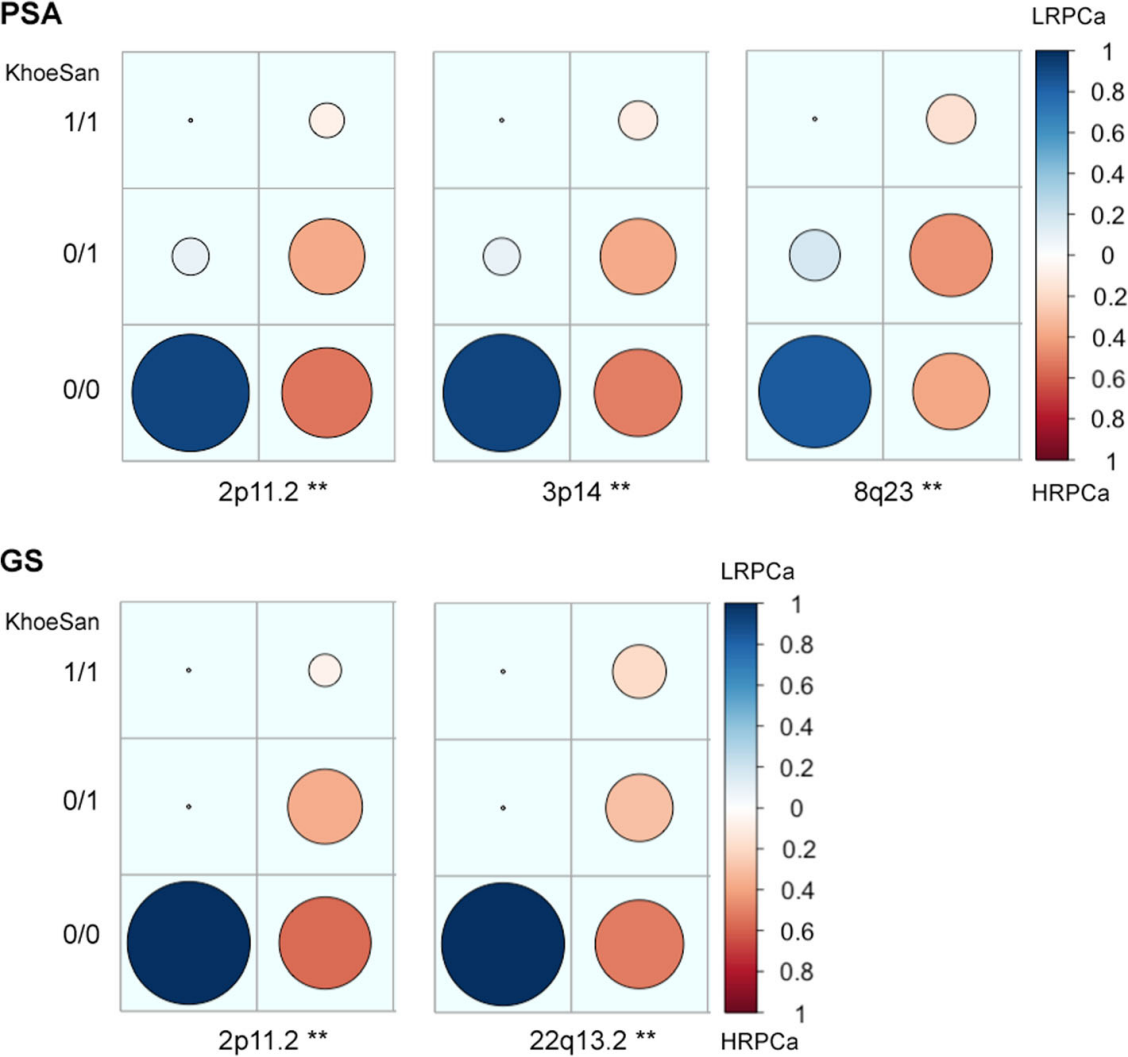
Candidate high-risk prostate cancer (HRPCa) chromosomal regions defined as an over-abundance of KhoeSan heritage. Legends show the proportion of Coloured participants presenting with HRPCa (red) versus low-risk prostate cancer (LRPCa; blue); asterisks (**) indicate regions with age-adjusted *P*-values < 0.01; 1/1, 0/1 or 0/0 represent the presence of KhoeSan ancestry within both DNA strands, a single strand or none, respectively. The local ancestry is defined using RFMix

Associating excess KhoeSan contribution within HRPCa presentation in the Coloured, we performed a local-ancestry inference analysis for KhoeSan-specific enrichment, using RFMix [15]. The most significant age-adjusted KhoeSan ancestral association with GS ≥ 8 was observed at chromosome 22q13.2 (95 markers; GRCh37 positions 40,178,619-42,552,253; ANOVA *P*-*value* = 0.0062) and chromosome 2p11.2 (332 markers; positions 80,741,406-85,833,046; ANOVA *P-value* = 0.0083) (Fig. 3). While KhoeSan ancestry was also associated with an elevated PSA ≥ 20 ng/ml at 2p11.2 (ANOVA *P-value* = 0.0004), two additional PSA-HRPCa associated loci were identified, including chromosome 3p14 (127 markers; positions 57,971,523-59,436,405; ANOVA *P-value* = 0.0026) and 8q23 (79 markers; positions 111,028,667 to 112,656,042; ANOVA *P-value* = 0.0052). Performing haplotype and single marker association test we identified two markers, rs10103786 and rs4504665, within 8q23 that remained significant after correcting for multiple testing (1000 permutations; Chi-Square = 15.365 and 11.245; *P*-*value* = 0.007 and 0.048, respectively).

## Discussion

HRPCa is the major contributor to prostate cancer mortality. The highest mortality rates globally are reported for the Caribbean (29.3 per 100,000), as well as southern, middle, western and eastern Africa (range 24.4 to 18.7 per 100,000) [18]. Asian countries, with overall elevated life expectancy, present with the lowest mortality rates (range 7 to 2.9 per 100,000). Controlling for geography, within the United States, African Americans are at 2.4- and 5-fold greater risk for prostate cancer mortality compared with Americans of European or Asian ancestry, respectively [19]. Elevated mortality rates reported across the Caribbean, United States and Africa, with further implications for familial history of disease as a significant risk factor, raise an important question regarding the contribution of African genetic ancestry to HRPCa.

We determined the contribution of African ancestral contributions defined as Bantu and KhoeSan to increased HRPCa presentation within South Africa. In contrast to African Americans, Black South Africans present with uniquely Bantu, specifically Southern over West Bantu or West non-Bantu contribution, with a single pulse KhoeSan contribution occurring over 550 years ago. The South African Coloured present, on average, with matched non-African to African genetic contributions. Interestingly, the non-African fraction includes both European and Asian contributions, representing intermediate-risk and low-risk populations for HRPCa. Specifically, the African initiating admixture event predates African American admixture by two generations and includes significant KhoeSan contributions followed to a lesser extent by Bantu contribution. We demonstrate that the South African Coloured represents a unique and alternative resource to African American studies for identifying significant African ancestral contributions to elevated HRPCa.

Confirming an African ancestral link to HRPCa within the Coloured, we showed that the observed significance appears to be driven largely by a KhoeSan over Bantu contribution. Although we must caution the need for replication due to a relatively small study size, to the best of our knowledge, this is the first report linking ancient KhoeSan ancestry and prognosis of a common modern condition. It would be reasonable to speculate that prostate cancer risk alleles would not be under negative selection within a hunter-gatherer society with an on average younger overall lifespan. Using KhoeSan ancestry as a surrogate for HRPCa, we have identified four chromosomal regions as potential risk loci for aggressive presentation within the region. The 2p11.2 locus, enriched for both GS ≥ 8 and PSA ≥ 20 ng/ml, has previously been associated with PCa risk [20, 21]. A recent study, using capture-based Chromosome Conformation Capture (3C) sequencing, identified a significant physical long-range interaction between common variants within the largely non-coding 2p11.2 region and the candidate tumor suppressor gene *CAPG,* with expression quantitative trait locus signals at rs1446669, rs699664 and rs1078004 (absent within our array content) [22]. Additionally, the GS-associated 22q13.2 region has previously been associated with HRPCa in a roughly 1000 strong Swedish genome-wide association study, with independent rs7291691 cross study validation. Located at position 38,778,569, the latter common variant is upstream of the region identified in this study, which may indicate a population specific impact [23]. Notably, the PSA-associated regions, 3p14 and 8q23, are both proximal to known prostate cancer risk loci, including a deletion of the 3p14.1-3p13 region in HRPCa [24] and the common 8q24 prostate cancer risk loci [22].

## Conclusions

In summary, this is the first study to link ancient KhoeSan ancestry to a common modern disease. Specifically, we link KhoeSan ancestry to HRPCa presentation within a uniquely admixed population with African, KhoeSan and Bantu, as well as non-African, European and Asian, ancestries. Using KhoeSan ancestry as a surrogate for HRPCa, we identify potential candidate loci, although one must caution that these regions are only suggestive and require larger study numbers to meet levels of genome-wide significance. However, previously two regions, 2p11 and 22q13 have been suggested as HRPCa risk loci, while two variants at 8q23 remained significant when accounting for multiple testing. Our findings suggest that modern humans earliest ancestors may have been carrying genomic signatures for HRPCa, which would not have been selected against due to later age of onset of prostate cancer. Although largely under-represented in contemporary populations, our study suggests a unique modern application to ancient KhoeSan genetic ancestry.

## Additional files

Additional file 1: **Table S1**. Clinical classification of Coloured patients with prostate cancer. (DOCX 13 kb)

Additional file 2: Genotyping data of 68 Black participants (PED and MAP formats). (ZIP 16703 kb)

Additional file 3: Genotyping data of 84 Coloured participants (PED and MAP formats). (ZIP 1230 kb)

## Data Availability

All data generated or analysed during this study are included in this published article and its supplementary information files (Additional file 2 and Additional file 3).

## Abbreviations

cM: centimorgan
GS: Gleason score
HRPCa: high-risk prostate cancer
LRPCa: low-risk prostate cancer
PSA: prostate specific antigen
SAPCS: Southern African Prostate Cancer Study
