# Supplementary material for: African KhoeSan ancestry linked to high-risk prostate cancer
Source: BMC Med Genomics. 2019 Jun 4;12:82. doi: 10.1186/s12920-019-0537-0 (PMC6549381; doi:10.1186/s12920-019-0537-0)
Supplement: Supplementary file 1 — Table S1. Clinical classification of Coloured patients with prostate cancer. (DOCX 13 kb) [file 12920_2019_537_MOESM1_ESM.docx]

**SUPPLEMETARY TABLE**

**Table S1 – Clinical classification Coloured patients with prostate cancer**

|  | **Total of 84 Coloured PCa patients** | |
| --- | --- | --- |
| **Clinical characteristic**  Age (years)  Serum PSA (ng/ml) | **Median** | **Range** |
|  | 70  19.6 | 53 – 94  3.2 – 4390 |
| **Mitochondrial haplogroup**  KhoeSan (L0d, L0k)  African (L0, L0a, L0f, L1, 2, 3, 4, 5)  Out of Africa (All other haplogroups) | **n = 84**  41  17  26 | **Percentage**  49%  20%  31% |
| **Gleason Score (GS) Group at diagnosis**  Low risk group, GS = 6  Intermediate risk group, GS = 7  High risk group, GS = 8, 9 and 10 | **n = 84**  12  45  27 | **Percentage**  14%  54%  32% |
